# The effect of erythropoietin on biomechanical properties of the Achilles tendon during the healing process: an experimental study

**DOI:** 10.1186/s13018-016-0390-1

**Published:** 2016-04-28

**Authors:** Okkes Bilal, Ahmet Guney, Ali Murat Kalender, Ibrahim Halil Kafadar, Muzaffer Yildirim, Nuh Dundar

**Affiliations:** Department of Orthopaedics and Traumatology, Sutcu Imam University Medical Faculty, Kahramanmaras, Turkey; Department of Orthopaedics and Traumatology, Erciyes University Medical Faculty, Kayseri, Turkey; Department of Pathology, The Ministry of Justice, Council of Forensic Medicine, Istanbul, Turkey

**Keywords:** Erythropoietin, Tendon, Healing, Tensile test, Biomechanical properties

## Abstract

**Background:**

The aim of this study was to examine the potential biomechanical and histological benefits of systemic erythropoietin administration during the healing of Achilles tendon injury in a rat experimental model.

**Methods:**

Eighty Sprague-Dawley female rats were included in this study. Animals were randomly assigned into two groups with 40 animals in each: erythropoietin group and control group. Then each group was further divided into four subgroups corresponding to four time points with 10 animals in each. A full-thickness cut was made on the Achilles tendon of each animal and then the tendon was sutured with modified Kessler method. Erythropoietin groups received intraperitoneal erythropoietin (500 IU/kg/day) every day at same time throughout the study period, and the control groups received saline in a similar manner. Animals were sacrificed at four time points, and tensile test was performed on each tendon sample to assess maximum load for each sample. In addition, histopathological examination and scoring was done.

**Results:**

Both groups had improvement on tensile test (maximum load) over time. However, groups did not differ with regard to maximum load in any of the time points. Similarly, groups did not differ with regard to any of the histopathological scores over time.

**Conclusions:**

The findings of this study do not support the benefit of systemic erythropoietin administration in Achilles tendon healing process. Further evidence from larger experimental studies is required to justify any such potential benefit.

**Electronic supplementary material:**

The online version of this article (doi:10.1186/s13018-016-0390-1) contains supplementary material, which is available to authorized users.

## Background

The tendons are strong structures that have to resist high tension loads, and healing of tendon injuries are of special concern in practice. Tendon healing is relatively a slower process when compared to other connective tissue structures, and the main goal of the treatment of tendon injuries is to restore mechanical strength. The Achilles tendon is the thickest and the strongest tendon in the body [1, 2], and it has important role in locomotion.

Erythropoietin is a 34-kD glycoprotein hormone that regulates the development of erythrocytes in the bone marrow through binding a high affinity receptor expressed on erythroid progenitor cells. Owing to its hematopoietic properties, erythropoietin has been used in the treatment of patients with chronic renal disease or chemotherapy-associated anemia. In addition, erythropoietin receptor is expressed on other cell types, suggesting other roles for this hormone. Further research identified its potential antioxidant [3], anti-apoptotic [4], anti-inflammatory [5], angiogenic [6], neuroprotective [7], and enhanced wound healing effects [8]. Its role in wound healing has been tested in many studies using different soft tissue injury models [8–11]. However, studies testing its role in the healing of tendon injuries are relatively scarce [12].

Tensile strength test is a useful test to examine biomechanical properties of tissues. During this test, the sample material is pulled and its ability to preserve its integrity is measured. A full tensile profile can be obtained when the test is continued until the failure of the material. Increased angiogenesis is a part of repair process of the injured tissues, and histological examination provides information on the level of revascularization during healing.

The aim of this study was to examine the role of systemic erythropoietin administration in the healing of an experimental Achilles tendon injury as assessed by tensile strength test and histopathological evaluation.

## Methods

### Experimental animals

Eighty Sprague-Dawley female rats weighing between 200 and 350 g were included in this study. The study was conducted at the Experimental Research Laboratory of Sutcu Imam University, Kahramanmaras, Turkey. The study protocol was approved by the local ethics committee for animal experiments of Kahramanmaras Sutcu Imam University Medical Faculty (reference number: 06.03.2013/02). The animals were kept at a standard animal care environment and fed with standard animal food. Animals were first randomly assigned into two groups with 40 animals in each: erythropoietin group (group E) and control group (group C). Then each group was further divided into four subgroups corresponding to four time points with 10 animals in each (groups E1 to E4 and groups C1 to C4).

### Surgical procedures

Subjects were anesthetized with intramuscular ketamine HCl 200 mg/kg (Ketalar^®^ Eczacibasi, Turkey) and xylazine 1 mg/kg (RompunR, Bayer, Turkey). A single dose of 0.1 mg/kg intramuscular cefazolin sodium (Cefozin, Bilim Ilac, Turkey) was administered for infection prophylaxis prior to surgical interventions.

**Fig. 1:**
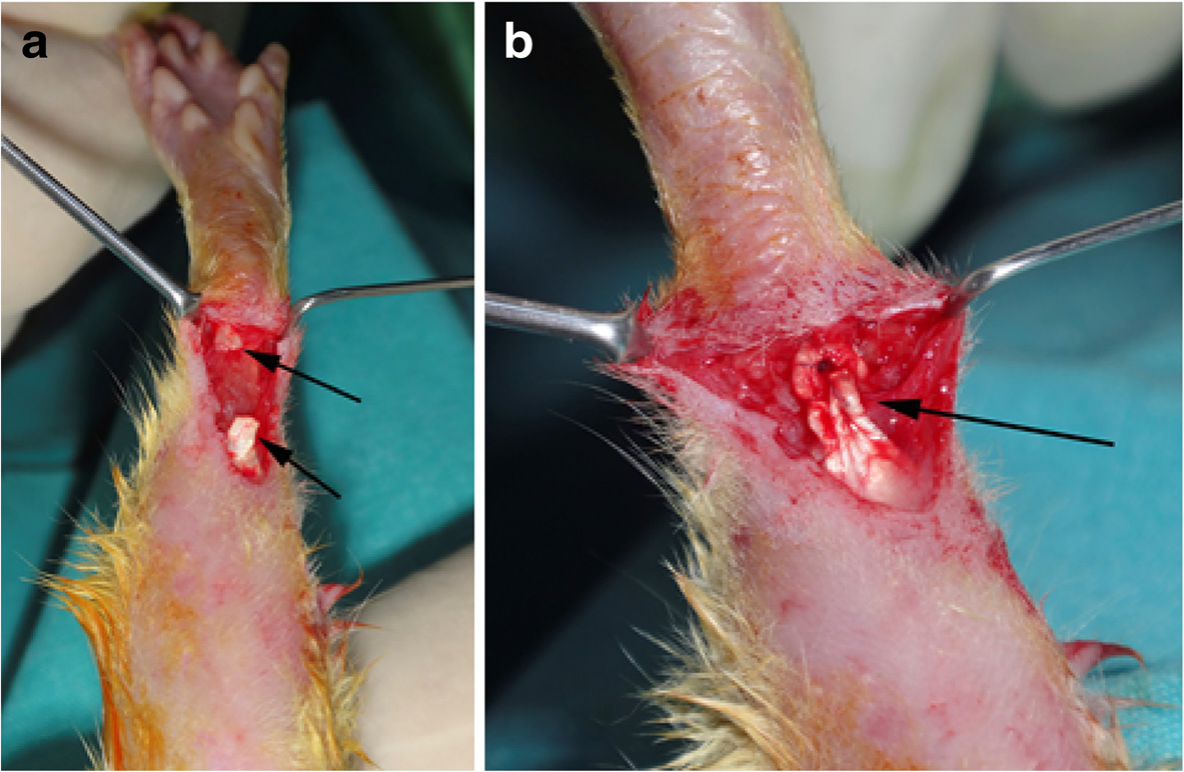
Achilles tendon repair technique. **a** *Arrows* indicate the two ends of the cut tendon; **b** the *arrow* points the site of tendon repair using modified Kessler method

The right hind legs of animals were shaved, betadine was applied, and surgical site was covered with a sterile dressing. A 3-cm incision was made over the Achilles tendon, and the peritendon was opened. A full-thickness cut was made on the Achilles tendon approximately 0.5 cm above its insertion to the calcaneus. The plantaris tendon was also cut to avoid splint effect. Then the Achilles tendon was sutured using 6/0 monofilament polydioxanone suture material (PDS^®^, Ethicon Inc., Bridgewater, NJ, US) with modified Kessler method. The skin was closed using 4/0 monofilament suture material (Prolene^®^, Ethicon Inc., Bridgewater, NJ, US). Figure 1 shows the technique for cutting and repair of the tendons. Rats were allowed to have free cage movement after surgery.

### Study interventions and sampling

The same investigator administered intraperitoneal erythropoietin (500 IU/kg/day) every day at same time throughout the study period to the rats in the erythropoietin group. Control group received the same volume of isotonic NaCl in a similar manner every day.

Ten animals from each treatment group (erythropoietin and controls) were sacrificed with high dose of pentobarbital anesthesia at weeks 1, 2, 3, and 4. The Achilles tendons of the subjects were removed, seven of them were used for biomechanical experiments and the remaining three were used for histopathological examination. Histopathological specimens were kept in formaldehyde solution until examination. The remaining specimens were kept at −80 ° C prior to biomechanical tests.

### Assessments

Tensile strength tests were done with ZWICK ROELL Z 5.0 device. In order to prevent tendon damage and slipping, non-slipping pads consisting of 2-mm thick rubber plates covered with sand paper were used to fix the tendons to the clamps with a cyanoacrylate adhesive (303-A Bruno^®^). Proximal and distal tips of the tendons were placed between the clamps with pads and they were tightened using six bars of pneumatic pressure. The distance between the clamps was adjusted to obtain a tendon length of 5 cm, and the rate of the device was set at 0.1 mm/s for the tensile test. In order to achieve an equal length and parallel alignment of the collagen fibers, a pre-loading under a standard load of 1 N was performed for 1 min prior to testing. Then, the tensile test was performed until tendon failure. TestXpert II software was used for data analysis, and a stress (applied force) versus strain (change in length) graph was constructed to estimate maximum load in Newtons for each sample.

Histological samples were taken from a plane adjacent to the suture site where macroscopically healing reaction was evident; and 3- to 5-μm thin sections were prepared after formalin fixation. Then preparations were stained with hematoxylin-eosin and Masson’s trichrome stains for examination under light microscope and assessments were done. Tissue revascularization, inflammation, fiber structures [13], and hyaline degeneration [13] were scored semi-quantitatively using a 0- to 3-point scale as defined in Table 1. In addition, a number of active fibroblasts were evaluated as follows: mesenchymal cells with irregular cytoplasm, ovoid and large light staining nucleus, and marked nucleolus were counted at three ×40 magnification fields and the mean of three measurements was used for analysis [14]. Histopathological examination was done by a single pathologist blinded to the study subjects.

**Table 1:** Scoring criteria for histological findings

	Score			
	0	1	2	3
Revascularization	None	Mild	Moderate	Significant
Inflammation (percent of inflammatory cells)	None	≤2 %	>2–≤10 %	>10 %
Fiber structures	None	Thin and sparse fiber structures	Irregular fiber structures	Regular fiber structures
Hyaline degeneration (percentage of areas with chondroid-like cells)	None	≤10 %	>10–≤30 %	>30 %

### Statistical analysis

SPSS version 21.0 was used for statistical analyses. Within-group comparisons of time points were done using Kruskal-Wallis test. For pairwise comparisons, built-in post hoc test of the statistical software for Kruskal-Wallis was used. For inter-group comparisons at each time point, Mann-Whitney *U* test was used. A *p* value <0.05 was considered an indication of statistical significance.

## Results

Table 2 shows tensile test results of the experiments (maximum load [load at tendon rupture]). Both groups had improvement over time. Week 4 (*p* = 0.001) and week 3 (*p* = 0.012) values were higher than week 1 value in the erythropoietin group. In controls, week 4 value was higher than week 1 value (*p* = 0.001) (Additional file 1). However, groups did not differ at any of the time points.

**Table 2:** Results of tensile test throughout the experiment for each study group

	Week 1	Week 2	Week 3	Week 4	*p*
Erythropoietin group	4.9 ± 2.9	10.6 ± 4.04	19.5 ± 7.8	19.9 ± 4.7	0.001
Control group	4.0 ± 2.0	8.2 ± 2.3	12.8 ± 3.5	20.2 ± 8.7	0.002
*P* for intergroup difference	1.000	0.329	0.167	1.000	

Data are presented as mean ± SD

In the erythropoietin group, week 4 (*p* = 0.001) and week 3 (*p* = 0.002) values are higher than week 1 value

In controls, week 4 value is higher than week 1 value (*p* = 0.001)

Raw data is provided in Additional file 1

**Fig. 2:**
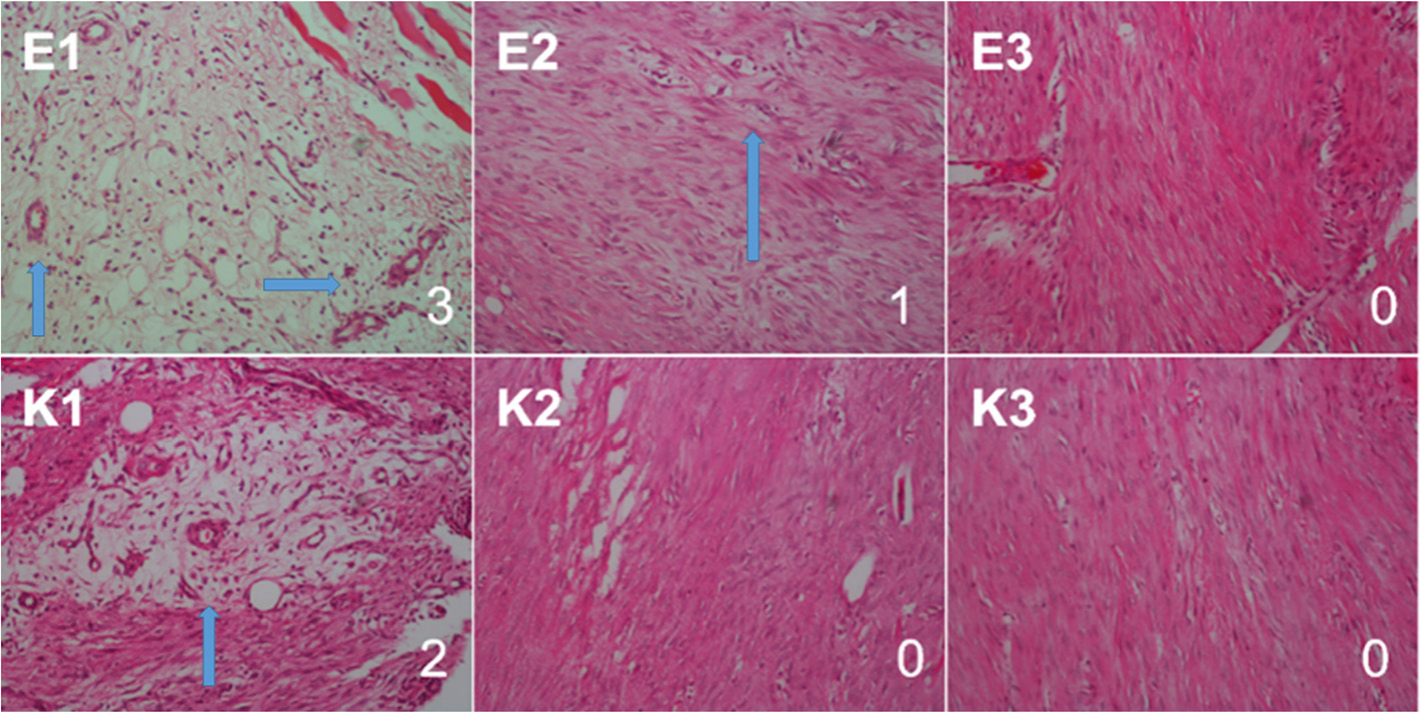
A representative serial revascularization images of the erythropoietin group (*E1* through *E3*) and the control group (*K1* through *K3*) at different time points (week 1 through week 3) with their semi-quantitative revascularization scores. Figures at the *right bottom corner* denotes revascularization scores. Hematoxylin and eosin stain, ×20 magnification. *Arrows* indicate vascular proliferation areas

Table 3 shows the changes in revascularization, inflammation, fiber structure, and hyaline degeneration scores and number of fibroblasts throughout the experiment for each study group (Additional file 2). Revascularization scores of the erythropoietin group showed a significant decrease throughout the experiment (*p* = 0.012) starting from the third week (*p* = 0.034) (week 4 (*p* = 0.034) and week 3 (*p* = 0.034) values were lower than week 1 value). Although the control group also showed a decrease in revascularization scores over time (*p* = 0.035), post hoc analysis did not identify a significantly different time point. Figure 2 shows representative serial revascularization images of the two groups at different time points with their respective semi-quantitative revascularization scores. Fiber structure scores significantly increased during study period in both erythropoietin group (week 4 > week 1, *p* = 0.025) and control group (week 3 > week, *p* = 0.034). Number of fibroblasts, hyaline degeneration scores, and inflammation scores did not change significantly throughout the study period in either of the groups. However, groups did not differ with regard to any of the histological scores at any of the time points (*p* > 0.05 for all).

**Table 3:** Changes in revascularization, inflammation, fiber structure, and hyaline degeneration scores and number of fibroblasts throughout the experiment for each study group

	Week 1	Week 2	Week 3	Week 4	*p* ^a^
Revascularization score					
Erythropoietin group	3 (3–3)	1 (1–1)	0 (0–0)	0 (0–0)	0.012
Control group	2 (1–2.5)	0.5 (0–0.5)	0 (0–0)	0 (0–0)	0.035^b^
*P* for intergroup difference	0.100	0.100	1.000	1.000	
Inflammation score					
Erythropoietin group	2 (2–3)	1 (0–1)	1 (0–1)	0 (0–1)	0.061
Control group	3 (2–3)	1 (0–1)	0 (0–1)	0.5 (0–1)	0.075
*P* for intergroup difference	0.700	1.000	0.700	0.800	
Fiber structure score					
Erythropoietin group	1 (0–1)	1 (1–1)	2 (2–2)	3 (3–3)	0.015
Control group	1 (1–1)	2 (2–2)	3 (3–3)	3 (3–3)	0.019
*P* for intergroup difference	0.700	0.100	0.100	1.000	
Hyaline degeneration score					
Erythropoietin group	0 (0–0)	1 (0–1)	1 (1–3)	2 (1–3)	0.051
Control group	0 (0–0)	1 (1–2)	3 (1–3)	2.5 (2–3)	0.053
*P* for intergroup difference	1.000	0.400	0.700	0.800	
Number of fibroblasts					
Erythropoietin group	22 (8–28)	40 (32–47)	54 (40–54)	27 (12–30)	0.030^b^
Control group	20 (16–33)	45 (27–52)	44 (30–45)	32 (30–35)	0.272
*P* for intergroup difference	1.000	1.000	0.400	0.200	

Data are presented as median (range)

^a^Overall *p* value for differences between four time points

^b^Despite statistical significance for overall difference, post hoc analysis did not identify a significantly different time point

Raw data is provided in Additional file 2

## Discussion

This study examined for the first time the potential benefit of systemic erythropoietin administration in the healing process of the Achilles tendon in a rat experimental model. Although, both erythropoietin and control groups showed biomechanical and histological improvement over time, erythropoietin group did not prove superior at any of the time points.

Erythropoietin is a glycoprotein hormone synthesized in the kidneys and acts mainly on erythroid progenitor cells. It has role in the control of erythropoiesis through its high affinity receptor (EPOR) expressed on its target cells. Besides erythroid cells, erythropoietin receptor (EPOR) has been found on other non-hematopoietic cells such as brain capillary endothelial cells [15], vascular smooth muscle cells [16], myocardial cells [17], neurons [18], and astrocytes [19]. In addition, erythropoietin is also produced in non-renal tissues such as the liver and brain [20]. Since it acts as a growth factor, it seems to stimulate mitosis and induce differentiation of cell lines, including endothelial, myocardial, smooth muscle, and mesangial cells. These properties stimulated research on its potential healing effects using different soft tissue models, with encouraging findings [8–11, 21, 22].

So far, a number of studies have examined the potential benefits of erythropoietin particularly in the healing of the injuries involving musculoskeletal system, most of them focusing on fracture healing [23–25]. In an experimental mice femur fracture model, a high dose of systemic erythropoietin (5000 U/kg/day for 6 days) was found to be associated with improved biomechanical properties and callus density after 2 weeks, when compared to controls [23]. These findings coincided with the expression of erythropoietin receptors in the terminally differentiating chondrocytes in the callus, suggesting a role for erythropoietin in early osteochondral ossification. Another experimental study examined the effect of a far lower dose of erythropoietin (500 U/kg/day)—similar to the present study—on the healing of femoral osteotomy and found improved biomechanical stiffness and density of the periostal callus after 2 and 4 weeks, compared to controls [25]. In addition, erythropoietin treatment was associated with higher endosteal vascularization and histologically more prominent bone formation. Similarly, in a rabbit mandibular distraction osteogenesis model, a similar systemic erythropoietin dose resulted in better vascularization and bone healing as evidenced by high number of osteoblasts and osteoclasts and a larger area of new bone formation [24]. In a recent clinical study, local injection of erythropoietin to fracture site resulted in shorter time to fracture union and lower non-union rate, indicating a potential role in accelerating bone healing process in human subjects [26]. Fewer studies examined the effect of erythropoietin on the healing of skeletal muscle healing. Rotter et al. administered 5000 IU/kg erythropoietin or saline to rats with experimentally traumatized left soleus muscle and assessed muscle recovery, microcirculation, tissue integrity, and cell proliferation over time, using muscle contraction capacity and immunohistochemical methods [27]. They found improved muscle strength, circulation, and cellular regeneration in erythropoietin-treated mice when compared to controls that received saline. In their experimental study, Jia et al. showed the role of erythropoietin signaling through non-hematopoietic receptors on skeletal myoblast proliferation and wound healing in a mouse model of cardiotoxin-induced muscle injury [28]. Mice with erythropoietin receptor restricted to hematopoietic tissue showed poor skeletal muscle healing response and higher susceptibility to skeletal muscle injury, indicating a role of erythropoietin signaling in skeletal muscle tissue apart from its role in the hematopoietic system.

Our literature search revealed only a single study examining the effects of systemic erythropoietin on tendon healing. In the experimental study by Uslu et al. [12], the rat patellar tendons were surgically cut and then repaired, and the biomechanical and histological effects of systemic erythropoietin administration (500 U/kg body weight, similar dose with the present study) were compared with controls (no treatment or saline administration). In that study, erythropoietin administration resulted in higher ultimate breaking force when compared to controls, both at 3 and 6 weeks, suggesting better tendon healing. Histopathological results were somewhat conflicting, since erythropoietin seemed to have a negative effect on fibroblast proliferation, capillary vessel formation, and local inflammation (as evidenced by lower scores reflecting less severe histopathological findings), but not on collagen organization at week 3. However, erythropoietin did not seem to have an effect on histological parameters on week 6. In contrast with these histological findings, erythropoietin administration was associated with higher tissue levels of collagen (Col) I, Col III, TGF-β1, and VEGF mRNA expressions. In that study, each experimental group had relatively small number of animals (*n* = 7), as it is the case in the present study. The present study on the other hand, could not demonstrate a beneficial effect of erythropoietin on Achilles tendon healing in our experimental model, although improved tendon strength was observed over time in both the study groups and controls.

A major concern regarding systemic erythropoietin treatment may be its possibly dose-related adverse effects. High systemic doses of erythropoietin may result in a prothrombotic state [29, 30], which may be particularly important and life threatening for patients with cardiovascular disease and for cancer patients. Thus, the optimal dose and treatment duration needs to be clarified. This study tested a relatively low systemic dose and did not show any beneficial effect on tendon healing, which is in contrast with the findings of several previous low-dose experimental studies. Although how experimental doses translate into benefits in clinical setting is another concern, further studies with higher but safe doses with varying treatment durations may reveal encouraging results.

Potential beneficial effects of erythropoietin on tissue healing may be attributed to its several physiological functions other than controlling hematopoiesis. Research has identified antioxidant [3], anti-apoptotic [4], anti-inflammatory [5], and angiogenic [6] properties, all may have potential role in tissue healing. Angiogenic properties have been attributed to a direct effect on VEGF production and endothelial cell mitosis [29, 31]. In addition to its angiogenic effects, it may modulate local environment at injury site through decreasing inflammation [32] and necrosis [33], recruiting stem cells [34] and modulating immune cell functions [35].

Regarding tendon injuries, local extracellular environment affects the process of tendon healing process, which is characterized by inflammation, fibroplasia, and remodeling phases. Tendon injury involves both disruption of vessels and alteration of the extracellular matrix. Inflammatory cell infiltration and migration, angiogenesis, tenocyte proliferation to produce collagen and matrix proteoglycan, and collagen reorganization takes place after tendon injury. Although non-hematopoietic properties of erythropoietin are expected to aid in better revascularization, fibrosis and ultimately in better tissue biomechanical properties, findings of this study did not support such an association. This may be due to the small sample size of this study, which is the main limitation, resulting in small number of rats at each time point, particularly for the assessment of histopathological changes. Alternatively, tendon tissue might differ from other soft tissues that benefit from erythropoietin at biochemical level, probably with regard to receptor expression. Nevertheless, based on studies with other tissues, erythropoietin seems to have some beneficial effects on healing and its effect on tendon healing process merits further investigation in trials with larger sample sizes. Experimental and clinical studies as well as molecular studies—for example, focusing on erythropoietin receptor expression on tenocytes—would shed light on this issue and may pave the way to helpful treatments for the healing of the repaired tendons, which represents a challenge in the clinical setting.

## Conclusions

Findings of this study do not justify the benefit of systemic erythropoietin administration in Achilles tendon healing process. Further evidence from larger experimental studies is warranted.

## Ethics approval

The study protocol was approved by the local ethic committee for animal experiments of Karamanmaras Sutcu Imam University Medical Faculty (date, March 6, 2013, No.,2).

### Consent for publication

Not applicable.

### Availability of data and materials

The datasets supporting the conclusions of this article are included within its additional files.

## Additional files

Additional file 1: Tensile test data. (XLSX 10 kb) Additional file 2: Data for pathological assessments. (XLSX 9 kb)

## Abbreviations

Col: collagen
EPOR: erythropoietin receptor
TGF-β1: transforming growth factor beta 1
VEGF: vascular endothelial growth factor
